# Plant nonsense-mediated mRNA decay is controlled by different autoregulatory circuits and can be induced by an EJC-like complex

**DOI:** 10.1093/nar/gkt366

**Published:** 2013-05-10

**Authors:** Tünde Nyikó, Farkas Kerényi, Levente Szabadkai, Anna H. Benkovics, Péter Major, Boglárka Sonkoly, Zsuzsanna Mérai, Endre Barta, Emilia Niemiec, Joanna Kufel, Dániel Silhavy

**Affiliations:** ^1^Agricultural Biotechnology Center, Institute for Genetics, Szent-Györgyi 4, H-2100, Gödöllő, Hungary, ^2^Gregor Mendel Institute, Austrian Academy of Sciences, Dr. Bohr-Gasse 3, 1030 Vienna, Austria, ^3^Albert-Ludwigs-Universitat Freiburg, Institut fur Biologie II/Botanik, Schanzlestrasse 1, D-79104 Freiburg, Germany and ^4^Institute of Genetics and Biotechnology, Faculty of Biology, University of Warsaw, Pawinskiego 5a, 02-106 Warsaw, Poland

## Abstract

Nonsense-mediated mRNA decay (NMD) is a eukaryotic quality control system that recognizes and degrades transcripts containing NMD *cis* elements in their 3′untranslated region (UTR). In yeasts, unusually long 3′UTRs act as NMD *cis* elements, whereas in vertebrates, NMD is induced by introns located >50 nt downstream from the stop codon. In vertebrates, splicing leads to deposition of exon junction complex (EJC) onto the mRNA, and then 3′UTR-bound EJCs trigger NMD. It is proposed that this intron-based NMD is vertebrate specific, and it evolved to eliminate the misproducts of alternative splicing. Here, we provide evidence that similar EJC-mediated intron-based NMD functions in plants, suggesting that this type of NMD is evolutionary conserved. We demonstrate that in plants, like in vertebrates, introns located >50 nt from the stop induces NMD. We show that orthologs of all core EJC components are essential for intron-based plant NMD and that plant Partner of Y14 and mago (PYM) also acts as EJC disassembly factor. Moreover, we found that complex autoregulatory circuits control the activity of plant NMD. We demonstrate that expression of suppressor with morphogenic effect on genitalia (SMG)7, which is essential for long 3′UTR- and intron-based NMD, is regulated by both types of NMD, whereas expression of Barentsz EJC component is downregulated by intron-based NMD.

## INTRODUCTION

Nonsense-mediated mRNA decay (NMD) is a eukaryotic surveillance system that identifies and degrades aberrant transcripts containing premature termination codons (PTC), thereby preventing the accumulation of the potentially harmful truncated proteins (1). NMD also regulates the expression of several wild-type transcripts, and the expression of 1–10% of the mRNAs is altered in NMD-deficient cells (2–4). NMD deficiency is lethal in plants, presumably because impaired NMD leads to constitutive pathogen response (4–7). NMD is also required for viability in *Drosophila* and vertebrates (8,9).

NMD directly targets (PTC containing and wild-type) transcripts harboring NMD *cis* elements in their 3′untranslated regions (UTRs). In yeast and invertebrates, unusually long 3′UTRs act as NMD *cis* element, whereas in vertebrates, 3′UTR-located intron(s) (positioned at least 50 nt from the stop) are the predominant NMD *cis* elements (10).

The NMD *trans* factors are partially conserved. The Up-frameshift (UPF)1, UPF2 and UPF3 NMD core factors are essential for both yeast and animal NMD. In animals, the suppressor with morphogenic effect on genitalia (SMG) factors that control the phosphoregulation of UPF1 are also required for NMD. Moreover, in vertebrates, the core components (Y14, Mago, Barentsz and eIF4AIII) of exon junction complex (EJC) are also involved in NMD (see later in the text) (11,12).

NMD is coupled to translation termination. NMD *trans* factors identify a stop codon as a PTC and initiates rapid degradation of the transcript if its translation termination is inefficient owing to the presence of NMD *cis* elements in the 3′UTR (13). For efficient termination, the eukaryotic release factor 3 (eRF3) component of the terminating ribosome has to bind to the poly(A) tail-binding protein (PABP). However, if an NMD *cis* element (for instance, an unusually long 3′UTR) inhibits the interaction between eRF3 and PABP, the eRF3 recruits the UPF1 NMD factor to the terminating ribosome (14,15). Then, the UPF1–UPF2–UPF3 functional NMD complex is formed, and the transcript degradation is initiated when the eRF3-bound UPF1 recruits UPF2 and UPF3 NMD factors. In yeast, formation of the UPF1–UPF2–UPF3 complex leads to decapping and rapid transcript decay, whereas in animals, the NMD complex triggers SMG1 kinase-mediated phosphorylation of UPF1. Phospho-UPF1 recruits three related 14-3-3-like domain-containing proteins (SMG5, 6 and 7) that induce decay of the transcript and dephosphorylation of UPF1. SMG6 cleaves the transcript close to the PTC, whereas the SMG5–7 heterodimer initiates a decapping-dependent exonucleolytic degradation pathway (16). In vertebrates, splicing of a 3′UTR intron located >50 nt downstream from the stop can dramatically intensify NMD. Vertebrate splicing leads to deposition of the EJC complex onto the mRNA 20–25 nt upstream of the exon–exon boundary. The core EJC forms a binding platform for the UPF3 and UPF2 NMD factors (17). The translating ribosome together with the associated Partner of Y14 and mago (PYM) protein removes EJCs from the 5′UTR and the coding regions but fails to dispose EJCs if it is bound to the 3′UTR >20–25 nt downstream of the stop (18). Thus, EJC–UPF3–UPF2 complex originating from the splicing of an intron located >50 nt downstream from the stop remain associated with the mRNA. The 3′UTR-associated EJCs increase the local concentration of UPF2 and UPF3 factors, thereby facilitating the formation of UPF1-2-3 NMD complex. Consequently, it enhances the efficacy of vertebrate NMD.

Although NMD is essential in plants, relatively little is known about plant NMD. Plants are the only eukaryotes in which both types of NMD *cis* elements, long 3′UTRs and 3′UTR-located introns can induce NMD efficiently (these two types of NMD are referred to as long 3′UTR-based and intron-based NMD, respectively) (3,19–21). We (and others) have shown that UPF1, UPF2 and SMG7 NMD factors are required for both types of plant NMD, and that UPF3 plays a role in long 3′UTR-based NMD (22–26).

Long 3′UTR-based plant NMD may be mechanistically similar to yeast and invertebrate NMD; UPF1 is recruited to the eRF3 component of the terminating ribosome if an unusual long 3′UTR inhibits the binding of eRF3 to PABP (26). It has been believed that the connection between splicing and NMD has evolved in vertebrates to eliminate the misproducts of alternative splicing (27,28). However, we showed that 3′UTR-located introns can also induce NMD in plants. Moreover, we have found that in plants, like in vertebrates, the 3′UTR introns trigger NMD in a position-dependent manner, and that homologs of two vertebrate EJC core components, Y14 and Mago, are required for intron-based plant NMD. Thus, we hypothesized that plant intron-based NMD, like vertebrate NMD, is mediated by an EJC-like complex (19,26). However, EJC has not been described in plants yet (29). Moreover, recent results that (i) in fission yeast, introns located nearby a stop codon (either upstream or downstream) stimulate NMD in an EJC-independent manner (30), (ii) in *Drosophila*, EJCs mark splicing sites differentially (31) and (iii) in mammals, not all splicing events lead to EJC deposition, whereas EJCs are frequently present on mRNAs in non-canonical positions (32,33), suggest that connections between splicing, EJC deposition and NMD are more complicated than expected.

As NMD regulates the expression of several wild-type mRNAs, it is not surprising that the intensity of NMD is strictly controlled. In mammals, the core NMD factors that are required for both types of NMD (SMG-1, −5, −6, −7, UPF1, UPF2, UPF3, but not the members of the EJC complex) are regulated by long 3′UTR-based NMD (34). Moreover, NMD sensitivity of these core NMD factors is tissue specifically changed (35). Thus, mammalian NMD is controlled by complex autoregulatory circuits. NMD is also subjected to developmental regulation in mammals. During neuron differentiation, NMD activity is gradually reduced, as miRNA128, which downregulates the expression of UPF1, is induced (36). NMD is also autoregulated in plants; it has been demonstrated that the mRNA of the SMG7 core NMD factor is directly targeted by NMD (4,26). However, it is not known if SMG7 mRNA is targeted by long 3′UTR-based and/or by intron-based NMD and that whether additional autoregulatory circuits are also involved in the control of plant NMD.

To better understand how splicing and NMD are coupled in plants, we wanted to further study the *cis* and *trans* factors of intron-based plant NMD and to analyze how plants control the intensity of the two NMD systems.

Here, we show that plant introns can trigger intron-based NMD if they are located >50 nt downstream of the stop codon, and that the efficiency of NMD is gradually increasing with the distance of the NMD inducing intron from the stop codon. We also demonstrate that orthologs of all four mammalian core EJC components (Y14, Mago, Barentsz and eIF4A3) are required for intron-based, but not for long 3′UTR-based NMD in plants. Moreover, we show that overexpression of PYM selectively impairs intron-based plant NMD. These results strongly support the hypothesis that intron-based plant NMD is mediated by an EJC-like complex. Interestingly, we found that plant NMD is controlled by different autoregulatory loops. Heterologous expression studies revealed that the transcript levels of *Arabidopsis* SMG7 NMD core factor are negatively regulated by both types of NMD, whereas the mRNA level of Barentsz NMD factor, which is involved only in intron-based NMD, is downregulated by the intron-based NMD. Conservation of the NMD-inducing sequence features of other angiosperm homologs of SMG7 and Barentsz suggests that these autoregulatory loops are conserved within angiosperms.

## MATERIALS AND METHODS

### Plasmid constructs

For agroinfiltrations, genes were cloned into Bin61S binary vector or into the derivatives of Bin61S (19,26). P14, GFP, G-600, U1DN, G-S7T, Y14DN, MDN, TRV-P, TRV-P-U1, TRV-P-U3, TRV-P-Y14, TRV-P-M, TRV-P-U2 and TRV-P-S7 clones were previously described. Other constructs are described in details at Supplementary Data S1. The list of primers is also shown at Supplementary Data S1.

### Agroinfiltration-based transient NMD assays

Agroinfiltration is the most efficient transient gene (co-)expression system for plants. To co-express different genes, each construct is introduced into an *Agrobacterium tumefaciens*, and then the different *Agrobacterium* cultures are mixed before infiltration. Agroinfiltration and GFP detection were described (19). Wild-type or silenced *Nicotiana benthamiana* leaves were agroinfiltrated with a mixture of bacterium cultures (OD_600_ of each culture was 0.4, or in the case of P14 0.2). Agroinfiltration induces RNA silencing, thus as a minimum, two bacteria are mixed, one expressed the P14 silencing suppressor, whereas the second bacterium expressed the test (or the control) construct (19). P14 also serves as internal control for western and northern assays. For dominant-negative assays, a third bacterium culture was added to the mix that expressed a dominant-negative mutant of UPF1, Y14 or Mago NMD factors (U1DN, Y14DN, MDN, respectively) (26). GFP fluorescence was detected by using a handhold long-wave ultraviolet lamp (UV products, Upland).

### Virus-induced gene silencing-agroinfiltration NMD assay

Virus-induced gene silencing (VIGS) is the most effective transient gene silencing system for plants. To silence a gene, a segment from that gene should be incorporated into a *Tobacco Rattle Virus* (TRV) VIGS vector, and then an *N. benthamiana* is infected with the recombinant VIGS vector. TRV infection induces strong antiviral RNA silencing response; thus, the virus concentration will be low in the upper leaves. Moreover, the silencing will specifically inactivate the host genes that are homologous to the incorporated sequence. To trigger VIGS, ∼21-days-old *N. benthamiana* plants were co-infiltrated with a mixture of three *Agrobacterium* cultures. One expressed P14, the second expressed TRV RNA1 and the third expressed TRV RNA2 containing segments from *N. benthamiana* phytoene-desaturase (PDS) or PDS+ ∼600 nt long segment from the gene what we wanted to silence (gene of interest). PDS was used to monitor silencing. The sequence from the gene of interest was cloned from reverse transcriptase-polymerase chain reaction (RT-PCR) fragments generated from *N. benthamiana* leaves. When the upper leaves started to bleach (indicating that PDS silencing was efficient and suggesting that the silencing of the gene of interest is also effective), leaves under the bleaching ones were agroinfiltrated with bacterium mixtures expressing P14 + a NMD reporter or a control construct (26,37).

### Protoplast transfection

Protoplast isolation and transfection were performed as described, with minor modifications. Protoplasts were isolated from 14-days-old *Arabidopsis thaliana* seedlings, transfected with plasmid DNA and incubated for 20–24 h at 22°C before RNA isolation (38).

### RNA gel blot and RT-PCR assays

RNA gel blot assays and quantifications were described (19). PCR fragments labeled with random priming method were used as probes. Phosphorimage measurements were used to quantify mRNA expression. RT-PCR was carried out with QIAGEN OneStep RT-PCR Kit.

For quantitative qRT-PCR studies, RNA was isolated using TriReagent (MRC). After DNAse I digestion, cDNA was reverse transcribed using random hexamer primers followed by qPCR with Sybr Select (Abi). RotorGene 6000 (Life Technologies) Real-Time PCR machine was used. Primers are listed in Supplementary Data S1.

## RESULTS

### Introns located >50 nt downstream from the stop codon can trigger NMD in plants

**Figure 1.**
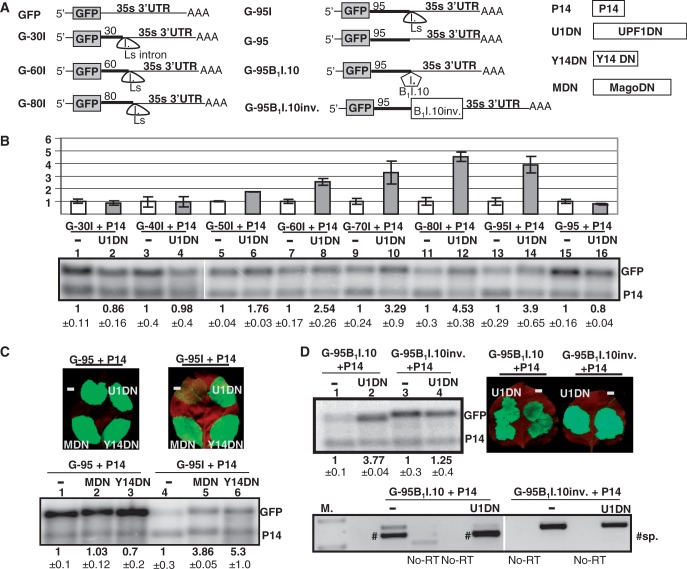
Introns located >50 nt downstream from the stop induce intron-based NMD in plants. (**A**) Schematic non-proportional representation of the used constructs. The NMD reporter constructs are shown as transcripts, whereas the co-expressed constructs are shown as proteins. The 35 s 3′UTR of the reporter transcripts is the 3′UTR of the 35S terminator. Note that not all NMD reporters are shown. (**B**) The Ls coding region intron cloned >50 nt downstream from the stop induces intron-based NMD. GFP-based NMD reporter constructs having 30–95 nt long stuffer sequences between the stop codon and the Ls intron (G-30I, G-40I, G-50I, G-60I, G-70I, G-80I and G-95I, respectively) and an intronless control construct (G-95) were co-infiltrated with the P14 silencing suppressor (−), or were co-infiltrated with P14 and a dominant-negative mutant of UPF1 (U1DN). P14 was co-infiltrated to suppress RNA silencing, and it also served as a normalization control for RNA gel-blot assays. Blots were hybridized with GFP and P14 probes. To quantify RNA samples, at each lane, the signal of the NMD reporter mRNA (GFP probe) was normalized to the corresponding P14 signal (GFP/P14 signal). Mean values were calculated from three independent samples. The GFP/P14 ratio of the (−) sample was taken as 1, and the GFP/P14 ratio of the U1DN co-infiltrated sample is shown relative to it (± shows standard deviation, SD). (**C**) The G-95I reporter transcript is exclusively targeted by intron-based NMD. The G-95I reporter construct and its intronless control (G-95) were infiltrated with P14 (−) or were co-infiltrated with P14 and with a dominant-negative mutant of UPF1, Y14 or Mago (U1DN, Y14DN and MDN, respectively). Strong fluorescence and the high transcript level at G-95 sample (−) indicates that the G-95 transcript is not targeted by NMD, whereas the weak fluorescence and the low mRNA levels at G-95I (−) sample show that the G-95I reporter transcript is a strong target of intron-based NMD. Enhanced fluorescence and increased transcript levels at G-95I + U1DN, G-95I + Y14DN and G-95I + MDN samples indicate that the co-infiltrated constructs inhibit intron-based NMD. Photos and RNA samples were taken at 3 days post inoculation (d.p.i.). (**D**) Genuine 3′UTR introns can trigger intron-based NMD. The 3′UTR intron of the putative *Arabidopsis* Barentsz1 (B_1_I.10) cloned 95 nt downstream from the stop (G-95B_1_I.10) triggers intron-based NMD, whereas its non-spliceable mutant version (G-95B_1_I.10inv) fails to induce NMD. Hash marks spliced transcripts (sp.). At the bottom panel, in the U1DN co-infiltrated sample, the spliced transcripts (hash marks) are overrepresented relative to the unspliced mRNAs.

Previously, we have shown that 3′UTR located introns trigger plant NMD in a position-dependent manner. The potato Ls intron cloned 90 nt downstream from a stop codon induced strong NMD, whereas the same intron inserted 20 nt downstream from the stop did not trigger NMD (19). To further study the role of intron position in NMD activation, a series of NMD test constructs were generated by cloning the Ls intron with 30, 40, 50, 60, 70, 80 or 95 nt long stuffer sequences between the GFP reporter gene and the 35S terminator (Figure 1A). NMD sensitivity of the test constructs was studied in agroinfiltration-based UPF1 dominant-negative (U1DN) co-expression assays (overexpression of U1DN, a dominant negative mutant form of UPF1 inhibits NMD) (19). The NMD test constructs were agroinfiltrated into *N. benthamiana* leaves only with a P14 RNA silencing suppressor or were co-infiltrated with P14 and U1DN (referred to as control and U1DN co-infiltrated samples, respectively). P14 was co-infiltrated in these experiments for two reasons: (i) to suppress agroinfiltration-induced intense RNA silencing (39) and (ii) to serve as an internal control for RNA gel blot assays. (Although P14 was co-infiltrated in all assays shown in this article, it will not be mentioned later in the main text. However, P14 is shown on the figures).

Expression of U1DN leads to the inactivation of NMD; therefore, NMD target transcripts accumulate to enhanced levels in U1DN co-infiltrated samples. As G-30I and G-40I transcripts accumulated to comparable levels in control and U1DN co-infiltrated samples (Figure 1B, compare lanes 1 with 2 and lanes 3 with 4), we concluded that these mRNAs are not targeted by NMD. By contrast, co-expression of U1DN significantly enhanced the transcript levels of G-50I, G-60I, G-70I, G-80I and G-95I mRNAs (Figure 1B), suggesting that these transcripts are targeted by NMD. Moreover, we found that transcripts with longer stuffer sequences accumulated to lower levels, and that U1DN co-infiltration enhanced their expression more dramatically (Figure 1B). Thus, we concluded that extension of the stuffer region from 50 to 80–95 nt gradually enhanced the efficiency of NMD. To exclude the possibility that the different NMD sensitivity of the reporter transcripts is the consequence of different splicing efficacy, RT-PCR assays were conducted. As expected, we found that the Ls intron was efficiently spliced from the reporter mRNAs, and that U1DN co-infiltration did not alter the efficiency of splicing (Supplementary Figure S2A).

To clarify whether these NMD target reporter transcripts are degraded exclusively by intron-based NMD or they also induce long 3′UTR-based NMD, an intronless control construct (G-95) was generated by deleting the Ls intron from G-95I. The G-95 control transcripts expressed to comparable levels in control and U1DN co-infiltrated samples (Figure 1B, compare lanes 15 with 16), suggesting that the insertion of a 95 nt long stuffer sequences into the 3′UTR do not induce long 3′UTR-based NMD. As the G-95 intronless control did not trigger NMD, whereas the G-95I transcripts, which after splicing are identical to the G-95 control mRNAs, were efficiently targeted by NMD (Figure 1B, compare lanes 13 and 14), we concluded that the G-95I mRNAs were exclusively targeted by intron-based NMD system. To further confirm that G-95I is targeted by intron-based NMD, it was co-expressed with dominant-negative mutant versions of Y14 and Mago (called Y14DN and MDN, respectively). Previously, we have shown that co-infiltration of Y14DN or MDN inhibits intron-based NMD but does not affect the long 3′UTR-based NMD (26). As expected, we found that co-infiltration of both Y14DN and MDN enhanced the transcript level of G-95I but did not modify the expression of G-95 transcripts (Figure 1C, compare lane 1 with 2 and 3, and lane 4 with 5 and 6). These data suggest that the Ls intron located ≥50 nt downstream from the stop can trigger NMD in plants, and that intron-based NMD acts gradually, the longer the distance from the stop, the more efficient the intron-induced NMD.

In these assays, the Ls intron from the coding region of potato patatin gene was used because it is efficiently spliced even in a heterologous 3′UTR context. As different mammalian introns trigger NMD with different efficiency (40), and not all splicing event results in EJC deposition (33), it is conceivable that 3′UTR plant introns, unlike the coding region Ls intron, do not induce NMD or activate NMD with low efficacy. To test whether a genuine 3′UTR intron can trigger plant NMD, we replaced the Ls intron of the G-95I construct with the 3′UTR intron of the *Arabidopsis* At1g80000 gene, one of the two closely related putative plant homologs of the Barentsz EJC factor (the intron is called B_1_I.10 for Barentsz1 intron 10, the construct is named G-95B_1_I.10). The B_1_I.10 intron was spliced relatively efficiently from the G-95B_1_I.10 transcripts (Figure 1D bottom panel). G-95 B_1_I.10 mRNAs accumulated to increased levels in the U1DN co-expressed sample relative to the control, suggesting that the G-95B_1_I.10 mRNAs are targeted by NMD (Figure 1D compare lanes 1 and 2). By contrast, the transcript level of the control construct (G-95 B_1_I.10inv) in which the B_1_I.10 intron could not be spliced as it was cloned in a reverse orientation, was not affected by the co-infiltration of U1DN (Figure 1D compare lanes 3 and 4). These results suggest that the B_1_I.10 3′UTR intron, like the Ls coding region intron, can trigger intron-based NMD. We found that other 3′UTR introns cloned into the same position (95 nt from the GFP) could also induce intron-based NMD, although their splicing efficacy and consequently their NMD-inducing effect varied (Supplementary Figure S2B). Thus, we concluded that spliceable plant introns located >50 nt downstream from the stop can activate intron-based plant NMD.

### UPF3 plays a role in intron-based plant NMD

Previously, we have shown that UPF1, UPF2 and SMG7 NMD core factors are required for both types of plant NMD, and that UPF3 plays a role in long 3′UTR-based NMD. However, we failed to prove that UPF3 is involved in the intron-based NMD (26). Taking advantage of the fact that the G-95I reporter construct is targeted exclusively by intron-based NMD, we used this construct to clarify whether UPF3 plays a role in the intron-based plant NMD.

**Figure 2.**
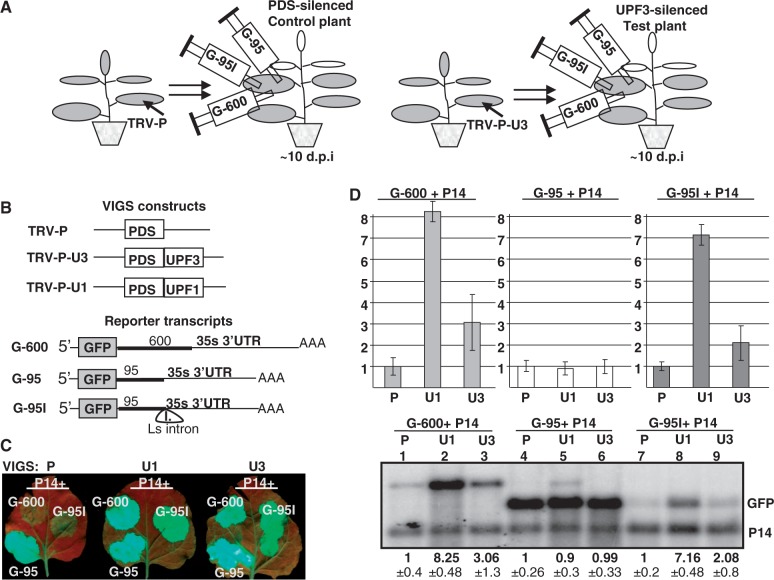
UPF3 is involved in both types of NMD. (**A**) The VIGS agroinfiltration NMD *trans* factor identification system. (**B**) Schematic non-proportional representation of the used constructs. TRV-P, TRV-P-U1 and TRV-P-U3 VIGS constructs were used to silence the control and the test genes. G-600 is the long 3′UTR-based NMD reporter, G-95I is the intron-based NMD reporter and G-95 is the non-target negative control construct. The NMD reporters and the control are shown as transcripts. (**C** and **D**) Both NMD reporter transcripts are overexpressed in UPF3-silenced plants. Leaves of PDS-silenced (P) negative control, UPF1-silenced (U1) positive control and UPF3-silenced (U3) test plants were infiltrated with P14 and with the G-600 or G-95I NMD reporters or with the G-95 control. Strong fluorescence and high transcript level of G-600 and G-95I infiltrated UPF1- and UPF3-silenced plants indicate that both types of NMD were impaired in these plants. RNA gel blots were hybridized with GFP and P14 probes, and then samples were quantified as described in Figure 1. The value of the PDS silenced plant (P) is taken as 1.

VIGS agroinfiltration NMD *trans* factor identification assay was used to study the role of the UPF3 in plant NMD (26,37). Briefly, VIGS was used to co-silence the UPF3 test gene and the PDS reporter gene in *N. benthamiana* plants. PDS co-silencing allows easy monitoring of the silencing efficiency because PDS silencing leads to whitening of the leaves. VIGS was also used to generate UPF1-PDS co-silenced positive control and PDS-silenced negative control plants (for simplicity, the UPF3-PDS and the UPF1-PDS co-silenced lines are referred to as UPF3- and UPF1-silenced plants, whereas negative control is called PDS-silenced plant). To study NMD activity in these silenced lines, PDS-, UPF1- and UPF3-silenced leaves were agroinfiltrated with the G-95I intron-based NMD reporter, the G-600 long 3′UTR-based NMD reporter and the G-95 non-NMD target control constructs (Figure 2A and B). If UPF3 is involved in both intron-based and long 3′UTR-based NMD, then both the G-600 and the G-95I reporter genes should be expressed to enhanced levels in UPF3-silenced plants compared with control PDS-silenced plants. Thus, the green fluorescence would be stronger, and the reporter transcript level would be higher.

As expected, the G-95 non-NMD target control constructs expressed to high and comparable levels in UPF3-silenced test, UPF1-silenced positive control and PDS-silenced negative control plants (Figure 2C and D, compare 2D lane 4 with 5 and 6). Moreover, confirming our previous result that UPF1 is required for both types of plant NMD (26), we found that both NMD reporter constructs accumulated to increased levels in UPF1-silenced plants relative to PDS-silenced negative control lines (Figure 2C and D, compare 2D lane 1 with 2 and lane 7 with 8). Relevantly, both NMD reporter constructs were overexpressed in UPF3-silenced plants relative to PDS-silenced control lines (Figure 2C and D, compare 2D lane 1 with 3 and lane 7 with 9). These data indicate that UPF3 plays a role in intron-based NMD and confirm our previous result that UPF3 is involved in long 3′UTR-based NMD (26). Notably, both NMD reporter constructs accumulated to higher levels in UPF1- than in UPF3-silenced plants (Figure 2D, compare lane 2 with 3 and lane 8 with 9). As silencing is never complete, we cannot distinguish between the alternative explanations that in *N. benthamiana*, UPF3 only facilitates NMD (while UPF1 is essential for NMD) or that UPF3 is also essential for NMD, but in the UPF3-silenced plants, the remaining UPF3 activity can partially fulfill its function.

To further confirm that UPF3 is involved in both types of NMD, protoplasts isolated from a UPF3 mutant (*upf3-1*) *Arabidopsis* were transfected with the G-95I intron-based NMD reporter, the G-600 long 3′UTR-based NMD reporter and the G-95 non-NMD target control constructs. As controls, protoplasts isolated form wild-type plant and from a hypomorhic UPF1 *Arabidopsis* mutant (*upf1-5*) were transfected with the same plasmids. The expression level of the reporter transcripts were studied by RNA gel blot assays at 1 day after transfection. In line with the results of VIGS agroinfiltration NMD assays (Figure 2), we found that both the G-600 and the G-95I reporter transcripts expressed to enhanced levels in the *upf3-1* and *upf1-5* protoplasts relative to the protoplasts that were isolated from wild-type negative control plants (Supplementary Figure S3A). The G-95I intron-based NMD reporter transcript and the control G-95 accumulates to comparable levels in *upf3-1* protoplasts, whereas the G-600 long 3′UTR-based NMD reporter transcript is not completely rescued in the *upf3-1* mutant. As *upf3-1* is supposed to be a null mutant, these results suggest that in *Arabidopsis*, UPF3 is required for intron-based NMD, and that UPF3 stimulates long 3′UTR-based NMD, but it is not absolutely essential for long 3′UTR-based NMD (24,41).

Taken together, we have shown that UPF3, like UPF1, UPF2 and SMG7 (Supplementary Figure S3), is involved in both types of plant NMD.

### The plant orthologs of core EJC components are required for intron-based but not for long 3′UTR-based NMD

**Figure 3.**
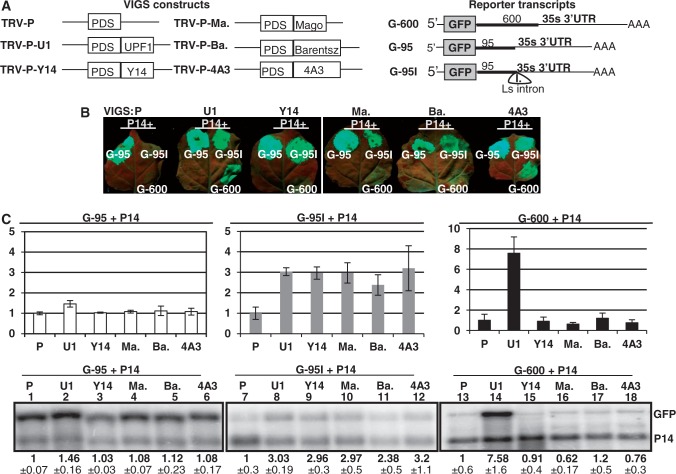
The plant homologs of the EJC components are required for intron-based NMD. (**A**) Schematic non-proportional representation of the different VIGS and the NMD reporter constructs. TRV-P, TRV-U1, TRV-Y14, TRV-Ma., TRV-Ba. and TRV-4A3 VIGS constructs were used to silence PDS, UPF1, Y14, Mago, Barentsz and eIF4A3 genes, respectively. (**B** and **C**) The G-95I intron-based but not the G-600 long 3′UTR-based NMD construct is overexpressed in Y14-, Mago-, Barentsz- and eIF4A3-silenced plants. PDS-silenced (P) negative control, UPF1-silenced (U1) positive control and Y14-, Mago-, Barentsz- and 4A3-silenced (Y14, Ma., Ba. and 4A3) test plants were agroinfiltrated with P14 and with the G-600 or the G-95I NMD reporters or with the G-95 control. RNA gel blots were hybridized with GFP and P14 probes, and then samples were quantified as described in Figure 1. Infiltration of G-600 reporter constructs into the eIF4A3-silenced (but not into Y14-, Mago- or Barentsz-silenced) leaves leads to moderately enhanced green fluorescence. However, G-600 transcript level is not enhanced in eIF4A3-silenced leaves, suggesting that eIF4A3 is not required for long 3′UTR-based NMD.

Previously, we demonstrated that Y14 and Mago play a role in intron-based but not in long 3′UTR-based plant NMD (26). If intron-based plant NMD is mediated by an EJC-like complex, the homologs of the eIF4A3 and Barentsz EJC core components should be also required for intron-based NMD. To test this assumption, VIGS agroinfiltration assays were conducted. The G-95I and the G-600 NMD reporter constructs (and the G-95 non-NMD target control construct) were expressed in eIF4A3-, Barentsz-, Y14- and Mago-silenced plants. UPF1-silenced plants were used as positive, and PDS-silenced plants were used as negative controls (Figure 3A). We found that (i) the G-95 control construct expressed to comparable levels in all plants, (ii) both NMD reporter transcripts accumulated to enhanced levels in the UPF1-silenced plants, (iii) the G-95I intron-based NMD reporter transcript was overexpressed in eIF4A3-, Barentsz-, Y14- and Mago-silenced plants relative to the PDS-silenced control and (iv) the G-600 long 3′UTR-based NMD reporter mRNA accumulated to comparable levels in PDS-silenced control and eIF4A3-, Barentsz-, Y14- and Mago-silenced test plants (Figure 3B and C). These results confirmed previous results that UPF1 is essential for both types of NMD and that Y14 and Mago are required only for intron-based NMD (26) and suggest that eIF4A3 and Barentsz are also required only for intron-based plant NMD. Thus, the plant orthologs of all four mammalian EJC core factors are required for intron-based NMD, but none of them are essential for long 3′UTR-based NMD. These data strongly support that in plants, like in mammals, an EJC-like complex couples splicing and NMD.

### Overexpression of PYM impairs intron-based plant NMD

**Figure 4.**
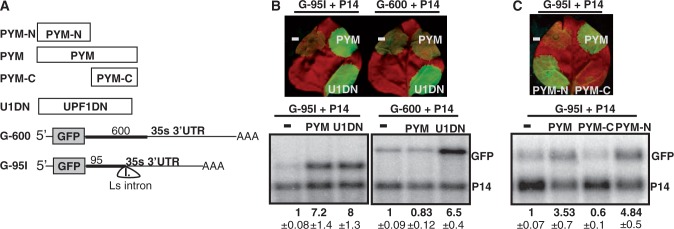
Overexpression of PYM inhibits intron-based NMD in plants. (**A**) Schematic non-proportional representation of the different NMD reporters and the co-infiltrated constructs. The NMD reporter and the control constructs are shown as transcripts, whereas the co-expressed constructs are shown as proteins. (**B** and **C**) Co-infiltration PYM or the N-terminal region of PYM inhibits intron-based NMD but does not interfere with long 3′UTR-based NMD.

In mammals, PYM acts as an EJC disassembly factor. In mammalian cytoplasm, PYM binds to the translating ribosomes via its C-terminal region and facilitates the removal of EJCs from the transcripts. Overexpression of PYM (or its EJC-binding N-terminal domain) impairs intron-based mammalian NMD, as free PYM can remove EJCs (including the NMD-inducing, 3′UTR located EJCs) from the mRNAs independently of translation (18). As PYM is a highly conserved protein and *Arabidopsis* PYM (At1g11400) also interacts with Y14 and Mago (42), we assumed that PYM also functions as an EJC disassembly factor in plants. If this assumption is correct, overexpression of PYM should inhibit intron-based but not long 3′UTR-based plant NMD. Indeed, co-infiltration of PYM led to enhanced expression of the G-95I intron-based NMD reporter mRNA but did not alter the expression the G-600 long 3′UTR-based NMD reporter transcript (Figure 4A and B). Moreover, we found that in plants, like in mammals (18), overexpression of the N-terminal PYM domain was sufficient to inhibit intron-based NMD (Figure 4C). These data suggest that PYM functions as an EJC disassembly factor in plants, and that it is involved in intron-based NMD. The molecular basis of the PYM action might be also conserved; we propose that the C-terminal region of PYM binds to the translating ribosome, whereas its N-terminal domain is involved in the disassembling of EJC.

### Barentsz expression is regulated by intron-based NMD in plants

**Figure 5.**
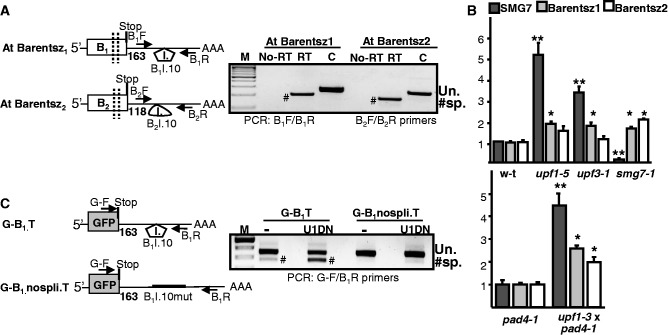
Barentsz mRNAs are targeted by NMD. (**A**) The 3′UTR introns are efficiently spliced from Barentsz1 and Barentsz2 mRNAs. Schematic non-proportional representation of *Arabidopsis* Barentsz1 and Barentsz2 transcripts (left panel). Only the 3′UTR located introns are shown. RT-PCR assays show that the 3′UTR introns (B_1_I.10, B_2_I.10) are efficiently spliced from both Barentsz1 and Barentsz2 transcripts in *Arabidopsis* leaves. Hash marks spliced transcripts. (**B**) Barentsz transcripts are overexpressed in NMD mutants. mRNA levels of Barentsz1 and Barentsz2 test genes were studied in wild-type (w-t) and in weak (*upf1-5*) or strong (*upf3-1*, *smg7-1*, *upf1-3*, *pad4-1*) NMD mutant *Arabidopsis* plants. The relative mRNA expression levels measured in the *upf1-5*, *upf3-1* and *smg7-1* lines are shown relative to the w-t plants, whereas relative transcript levels of *upf1-3*, *pad4-1* double mutant line are shown relative to the *pad4-1* sample. The *upf1-3* mutant is only viable in *pad4-1* background. Ubiquitin transcript was used as a normalization control. To prove that the NMD is inhibited in the mutants, the transcript level of SMG7 (S7) NMD sensitive positive control gene was used. Transcript levels were measured by qRT-PCR assays. (**C**) 3′UTR of Barentsz1 triggers intron-based NMD in heterologous assays. Schematic representation of the reporter transcripts (left panel). The terminator region of Barentsz1 (from the stop to 200 nt downstream of the polyadenylation site) was cloned downstream of the GFP reporter gene (G-B_1_T). As a control, a non-spliceable mutant version was created (G-B_1_nospli.T). G-B_1_T and G-B_1_nospli.T reporter construct were infiltrated with P14 or were co-infiltrated with P14 and a dominant-negative mutant of UPF1 (U1DN) into the leaves of *N.benthamiana* plants, and at 3 d.p.i., RT-PCR assays were conducted. The spliced forms (hash marks) of the G-B_1_T mRNAs are overrepresented in the U1DN co-infiltrated sample.

In addition to eliminating PTC containing aberrant mRNAs, NMD also directly regulates the expression of several wild-type transcripts having NMD *cis* elements (3,43,44). Thus, the intensity of NMD might be strictly controlled. Previously, we have shown that plant NMD is autoregulated, as the transcript of the SMG7 core NMD factor is targeted by NMD (26). To identify further potential autoregulatory circuits, we searched the sequences of the EJC components for NMD *cis* elements. In *Arabidopsis*, Y14 (At1g51510), Mago (At1g02140) and eIF4A3 (At3g19760) are single copy genes without obvious NMD features, whereas Barentsz is present in two closely related copies (Barentsz1-At1g80000 and Barentsz2-At1g15280). Relevantly, both Barentsz transcripts are potential targets of intron-based NMD, as their last intron (intron 10) is located in the 3′UTR 163 (Barentsz1) and 118 nt (Barentsz2) downstream from the stop (B_1_I.10 and B_2_I.10 intron, respectively). RT-PCR assays confirmed that both introns are efficiently spliced in *Arabidopsis* leaves (Figure 5A). To test whether Barentsz transcripts are downregulated by NMD, mRNA levels of Barentsz1 and Barentsz2 were compared in wild-type *Arabidopsis* plants as control and in *upf1-5* weak and *upf3-1*, *smg7-1* and *upf1-3*, *pad4-1* strong NMD mutant *Arabidopsis* lines (the *upf1-3* mutant is embryo lethal owing to constitutive expression of pathogen response genes, thus the *upf1-3*, *pad4-1* double mutant should be used, in which the pathogen response is inactivated) (6,41). The qRT-PCR studies revealed that Barentsz1 transcripts were significantly overexpressed in all NMD-deficient mutants, whereas Barentsz2 transcript levels were significantly upregulated in the strong mutants (except *upf3-1*, in which the Barentsz2 transcript level is slightly, but not significantly, enhanced) relative to the controls (Figure 5B). These data strongly indicate that in *Arabidopsis*, both Barentsz1 and Barentsz2 transcripts are negatively regulated by NMD.

To further study whether Barentsz1 transcripts can be directly targeted by NMD, the terminator region of Barentsz1 (the sequence from the stop to 200 nt downstream of the annotated Barentsz1 polyadenylation site) was cloned downstream of a GFP reporter construct (G-B_1_T), and then the NMD sensitivity of the G-B_1_T construct was studied in U1DN co-expression assay in *N.benthamiana* leaves. Unexpectedly, we found that U1DN co-expression did not lead to significantly enhanced G-B_1_T transcript levels (Supplementary Figure S4B). To distinguish between alternative explanations that (i) splicing of the B_1_I.10 intron from the Barentsz1 3′UTR does not induce NMD and that (ii) B_1_I.10 is not spliced from the G-B_1_T transcript in *N. benthamiana*, we studied the splicing efficiency in RT-PCR assays. We found that the B_1_I.10 intron was inefficiently spliced from the G-B_1_T transcript (Figure 5C). However co-infiltration of U1DN resulted in enhanced accumulation of the PCR products that correspond to the spliced transcripts (Figure 5C). Thus, we concluded that although the splicing of the B_1_I.10 intron from the Barentsz1 3′UTR context is inefficient in *N. benthamiana*, when it occurs, it can trigger intron-based NMD. To prove that the unspliced transcripts are not targeted by long 3′UTR-based NMD, we generated a control construct in which the 3′ splice site is mutated (G-95B_1_nospli.T); thus, the B_1_I.10 intron could not be spliced (Figure 5C). As the G-95B_1_nospli.T transcript levels were comparable in U1DN co-infiltrated and control samples, we concluded that if the intron is not spliced form the 3′UTR of Barentsz1, the extended 3′UTR fails to trigger NMD (Supplementary Figure S4). This is not an unexpected finding because the B_1_10 intron is only 80 nt long.

Taken together, our data indicate that the Barentsz1 3′UTR induces intron-based NMD if its 3′UTR located B_1_I.10 intron is spliced and suggest that the efficient splicing of B_1_I.10 intron in *Arabidopsis* leaves subject the Barentsz1 transcripts to intron-based NMD. Thus, the 3′UTR of Barentsz1 transcript can ‘sense’ the intensity of intron-based NMD. We propose that a homeostatic control mechanism regulates intron-based NMD in *Arabidopsis*; the expression of the Barentsz1 (and perhaps the Barentsz2) component of the intron-based NMD system is negatively regulated by intron-based NMD.

### SMG7 3′UTR induces both long 3′UTR-based and intron-based NMD

**Figure 6.**
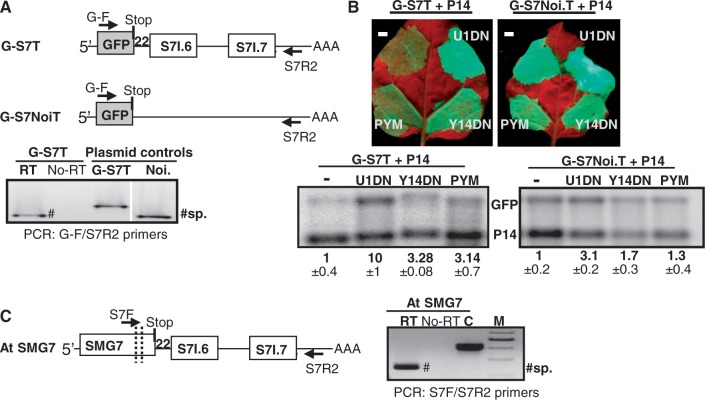
NMD sensitivity of the SMG7 3′UTR. (**A**) SMG7 3′UTR introns are efficiently spliced in heterologous context. Schematic non-proportional representation of the SMG7 3′UTR reporter transcript (upper panel). The terminator region of *Arabidopsis* SMG7 (from the stop to 200 nt downstream relative to the polyadenylation site) was cloned downstream of the GFP reporter gene (G-S7T). To evaluate the role of introns in NMD sensitivity, an intronless version of SMG7 terminator was also created and cloned downstream of GFP (G-S7Noi.T). RT-PCR shows that both SMG7 3′UTR located introns, the stop proximal S7I.6 and the distal S7I.7, are efficiently spliced from the agroinfiltrated G-S7T reporter mRNA. (**B**) SMG7 3′UTR triggers both long 3′UTR-based and intron-based NMD. G-S7Noi.T and G-S7T were infiltrated with P14 (−) or were co-infiltrated with P14 and PYM (PYM) or with P14 and a dominant-negative mutant of UPF1 or Y14 (U1DN, Y14DN, respectively). Both reporter mRNAs are overexpressed in the U1DN co-infiltrated samples, whereas the G-S7T, but not the G-S7Noi.T reporter transcript, is overexpressed when the intron-based NMD system is inhibited (Y14DN or PYM co-infiltrated samples). (**C**) *Arabidopsis* SMG7 3′UTR introns are efficiently spliced in *Arabidopsis* leaves. Schematic non-proportional representation of the SMG7 transcript (left panel). Only the 3′UTR located introns are shown. RT-PCR confirms that both 3′UTR introns are efficiently spliced in *Arabidopsis* leaves.

Previously, we have shown that SMG7 transcripts are direct targets of plant NMD (26). The 3′UTR of the SMG7 is unusually long and contains two introns (37). To clarify whether the SMG7 3′UTR is targeted by intron-based and/or by long 3′UTR-based NMD, the terminator of the *Arabidopsis* SMG7 was cloned downstream of the GFP reporter gene (G-S7T), and then the NMD sensitivity of the G-S7T reporter construct was studied in U1DN, Y14DN and PYM co-expression studies (Figure 6A and B). U1DN co-expression inhibits both types of NMD, whereas co-infiltration of PYM or Y14DN interferes only with intron-based NMD. The G-S7T transcript levels were strongly enhanced in U1DN co-expressed samples, confirming that SMG7 3′UTR triggers NMD. Relevantly, G-S7T mRNA was also overexpressed in PYM and Y14DN co-expressed samples relative to the controls, suggesting that SMG7 3′UTR activates intron-based NMD (Figure 6B). RT-PCR assays showed that both SMG7 3′UTR introns were as efficiently spliced from G-S7T mRNA in *N. benthamiana* as from the wild-type SMG7 transcript in *Arabidopsis* (Figure 6A and C). Thus, we concluded that splicing of the 3′UTR introns of SMG7 triggers intron-based NMD.

To test whether the 3′UTR of SMG7 can also induce long 3′UTR-based NMD, an intronless version was generated from the SMG7 terminator and cloned downstream of GFP (G-S7Noi.T). As Figure 6B shows, the intronless G-S7Noi.T transcripts accumulated to enhanced levels in U1DN co-infiltrated sample relative to the control, suggesting that the 3′UTR of SMG7 is long enough to induce long 3′UTR-based NMD (Figure 6B). Thus, the 3′UTR of SMG7 can sense the intensity of both types of NMD. Our data suggest that the expression of the SMG7 NMD factor, which is required for both types of plant NMD, is negatively regulated by both long 3′UTR-based and intron-based NMD.

In both *Arabidopsis* and rice, the 3′UTR of SMG7 contains two introns, the first intron is too close to the stop to induce NMD, whereas the distal intron is in an NMD relevant position. The surprising feature of the SMG7 3′UTR is the strong conservation of the stop proximal intron (37), which in theory is not required for the induction of intron-based NMD. Splicing of an intron could depend on the splicing of another intron. We hypothesized that the stop proximal intron is conserved because it is essential for the splicing of the distal intron. To clarify whether the splicing of the SMG7 3′UTR introns are co-dependent, new reporter constructs (G-S7Noi.6T, G-S7Noi.7T) were generated from G-S7T by deleting either the stop proximal or distal 3′UTR intron (SMG7 intron 6 and 7 are referred to as S7I.6 and S7I.7 intron, respectively) from the G-S7T constructs (Supplementary Figure S5). To study the splicing efficiency of the S7I.6 and S7I.7 introns, *N. benthamiana* leaves were infiltrated with G-S7Noi.6T and G-S7Noi.7T test and with G-S7T positive and G-S7Noi.T negative control constructs. RT-PCR assays showed that as expected, both introns were efficiently spliced from the G-S7T transcript (Supplementary Figure S5A). Moreover, we found that the S7I.6 intron was also efficiently spliced in the absence of the distal intron (G-S7Noi.7T transcript), suggesting that splicing of the stop proximal S7I.6 intron does not depend on the splicing of the distal intron (Supplementary Figure S5B). Relevantly, the S7I.7 intron was inefficiently and incorrectly spliced in the absence of stop proximal intron (Supplementary Figure S5C). This result indicates that splicing of the stop proximal intron of SMG7 is required for the correct and efficient splicing of the distal intron and that splicing of the distal intron triggers intron-based NMD.

Taken together, our data indicate that splicing of the S7I.7 intron subject the SMG7 transcript to intron-based NMD, and that the efficient splicing of the S7I.7 intron is depended on the splicing of the stop proximal S7I.6 intron. Consequently, both SMG7 3′UTR introns are required to trigger intron-based NMD. These results can explain the strong conservation of the unusual 3′UTR structure of the SMG7 transcripts within angiosperms.

## DISCUSSION

In this study, we analyzed the mechanism and regulation of intron-based NMD in plants. Our data suggest that in plants, like in vertebrates, an EJC-like complex couples splicing and NMD and that a complex autoregulatory system controls the intensity of plant NMD.

### The role of EJC-like complex in plant NMD

Long 3′UTRs induce NMD in plants, fungi, invertebrates and (less efficiently) in vertebrates, whereas 3′UTR located introns act as efficient NMD *cis* elements only in vertebrates and higher plants. Two eukaryotic NMD evolution models have been proposed. One model assumes that the long 3′UTR-based NMD is the evolutionary ancient form, and intron-based (EJC coupled) NMD has evolved in vertebrates to efficiently eliminate the misproducts of alternative splicing (27). An alternative model suggests that in stem eukaryotes (the last common ancestors of extant eukaryotes), both types of NMD were functional, and that intron-based NMD was already mediated by EJC in stem eukaryotes (19,45). This latter model predicts that EJC also plays a critical role in intron-based NMD in plants.

Our data strongly support the second model, indicating that in plants, like in vertebrates, intron-based NMD is mediated by an EJC-like complex. (i) As vertebrate EJC is deposited 20–25 nt upstream from the exon–exon boundaries and ribosomes can still displace EJC from the 20–25 nt long region downstream of the stop codon, NMD inducing vertebrate introns are located >50 nt downstream of the stop codon. Similarly, we found that 3′UTR introns located at least 50 nt downstream from the stop induce NMD in plants, suggesting that plant NMD is also triggered by a protein complex placed 25 nt upstream of exon–exon junctions. (ii) Orthologs of all four EJC core components are required for intron-based but not for long 3′UTR-based plant NMD. (iii) In plants and in mammals, overexpression of the PYM EJC disassembling factor (or its N-terminal region) inhibits intron-based NMD but did not affect on long 3′UTR-based NMD. (iv) In vertebrates, EJC plays a critical role in intron-based NMD by forming a binding platform for UPF3 and UPF2. We found that UPF3 and UPF2 are also involved in intron-based plant NMD. It is possible that in plants, like in vertebrates, 3′UTR located EJC facilitates NMD by binding UPF2 and UPF3.

These results and other indirect evidence, for instance, co-localization of putative plant EJC factors and conserved interactions among the components (Y14-Mago, PYM-Y14-Mago), strongly suggest that splicing leads to the formation of an EJC in plants (29,42,46–48), and that the 3′UTR-bound plant EJCs can trigger NMD. Thus, we propose that intron-based NMD is evolutionary conserved, and that a similar EJC-mediated intron-based NMD system has already functioned in stem eukaryotes.

Although our data suggest that the plant and vertebrate intron-based NMD operates similarly, it is likely that the mechanistic details are different. For instance, vertebrate NMD is limited to the pioneer round of translation because the CBP80 component of the CBP20-80 pioneer cap binding complex stimulates at least two steps of NMD, the recruitment of UPF1 to terminating ribosome and formation of the functional NMD complex (49). By contrast, the CBP factors are not required for plant NMD (50). These (and other) differences might lead to relevant biological consequences, for instance, that intron-based NMD acts more gradually in plants than in vertebrates, thus intron-based NMD might be more suitable for fine tuning of gene expression in plants (also see later in the text).

### Complex autoregulation of plant NMD

In plants, unlike in fungi or animals, both the long 3′UTR-based and the intron-based NMD systems act efficiently. Thus, the regulation of plant NMD might be especially complex. Indeed, we have identified two autoregulatory circuits that might provide partially separated homeostatic regulation for the two types of NMD in plants.

The mRNAs of SMG7 and Barentsz NMD factors are significantly overexpressed in *Arabidopsis* NMD mutants. Results that NMD *cis* elements are present in the 3′UTRs of the SMG7 and Barentsz transcripts and that in heterologous assays, both the SMG7 and Barentsz1 3′UTRs trigger NMD (although Barentsz1 3′UTR induced NMD inefficiently owing to the ineffective splicing), suggest that SMG7 and Barentsz1 mRNAs are direct targets of NMD. Interestingly, we found that the 3′UTR of the SMG7 core NMD factor, which is required for both types of NMD, can sense the efficiency of both types of NMD, whereas the 3′UTR of the intron-based NMD component Barentsz1 can sense only the intensity of the intron-based NMD. We propose that these two autoregulatory circuits allow partially separated fine tuning of the two NMD systems in *Arabidopsis* (Supplementary Figure S6).

If these autoregulatory systems play an important role in the fine-tuning of NMD, they should be conserved. Indeed, the structure (but not the sequence) of the SMG7 3′UTRs is highly conserved. All annotated angiosperm SMG7 transcripts (35 mRNAs, 6 monocot and 29 dicot transcripts) have NMD *cis* elements in their 3′UTR, 30 have long 3′UTR with two introns (an NMD-irrelevant proximal and an NMD-relevant distal intron), two have long 3′UTR with only NMD relevant intron(s) and three have long 3′UTR without introns (Supplementary Table S7A and B). Thus, the expression of 32 of 35 SMG7 transcripts could be sensitive to the activity of both types of NMD. By contrast, the mRNAs of the SMG7L homolog of SMG7, which is not required for NMD and is present only in dicots, do not contain NMD *cis* elements, all SMG7L transcripts (11 mRNAs) have short intronless 3′UTRs (Supplementary Table S7A and C) (23,37).

The strong conservation of the SMG7 3′UTR structure within angiosperms suggests that the capacity of SMG7 3′UTR to sense the intensity of both types of NMD is physiologically important. Our results that the stop proximal S7I.6 intron of *Arabidopsis* SMG7 is required for the efficient splicing of the NMD-relevant S7I.7 intron explains why this unusual 3′UTR intron structure (an NMD irrelevant proximal and an NMD relevant distal intron) is conserved within angiosperms. As both introns are required for efficient induction of intron-based NMD, the loss of either intron could lead to the loss of sensitivity to the intron-based NMD.

Transcripts of the Barentsz EJC factor (but not the transcripts of Y14, Mago or eIF4A3) contain NMD *cis* elements. In *Arabidopsis*, both Barentsz paralogs harbor an efficiently spliced NMD relevant intron (163 and 118 nt downstream from the stop). Importantly, the structure of Barentsz 3′UTR is also conserved, and 38 of 42 annotated angiosperm Barentsz mRNAs (Barentsz is a multicopy gene in most plants) contain at least one NMD relevant 3′UTR intron. Thus, almost all angiosperm Barentsz transcripts are potential targets of intron-based NMD (Supplementary Table S7A and D). Although, the number and the position of the Barentsz 3′UTR introns is not completely conserved, the strong conservation of the presence of NMD relevant intron(s) in the 3′UTRs suggests that the intron-based NMD-Barentsz autoregulatory circuit is physiologically important. As several Barentsz mRNAs contain 2–3 introns in the 3′UTR, it is possible that Barentsz expression is more sensitive to intron-based NMD in these plants than in *Arabidopsis*. Notably, several Barentsz mRNAs have relatively long 3′UTR; thus, we cannot exclude that certain Barentsz transcript sense the intensity of both types of NMD (Supplementary Table S7A and D). Moreover, the Barentsz 3′UTRs are subjects of alternative splicing, suggesting that the NMD sensitivity of these mRNAs might be regulated by alternative splicing.

The SMG7 mRNAs are targeted by NMD in plants and in animals, suggesting that the SMG7-NMD autoregulatory system is ancient; presumably, it was already functional in the stem eukaryotes (4,26,51). As the Barentsz–NMD autoregulatory circuit is plant specific, it might have evolved in the plant lineage before the dicot–monocot separation.

NMD activity is autoregulated in most (if not in all) eukaryotes. Two things might be specific for NMD autoregulation in plants, (i) intron-based NMD plays an essential role in autoregulation of NMD and (ii) a separate autoregulatory circuit might specifically control the efficiency of intron-based NMD through an EJC factor. In animals, transcripts of SMG7 (and mRNAs of several other NMD factors including UPF1, UPF2, UPF3, SMG1, SMG5 and SMG6) are direct targets of long 3′UTR-based NMD, whereas intron-based NMD does not play a role in the autoregulation of NMD. The EJC components are not controlled by NMD in vertebrates (34,35). By contrast, in plants, both SMG7 and Barentsz are downregulated by intron-based NMD. In vertebrates, intron-based NMD is extremely efficient and acts only during the pioneer round of translation (52); thus, it may not be used for fine tuning of gene expression. It might explain why only the less efficient long 3′UTR-based NMD is involved in the autoregulation of vertebrate NMD. As EJC factors are not required for long 3′UTR-based NMD (53), it is not surprising that their expression is not regulated by NMD. By contrast, in plants, both long 3′UTR-based and intron-based NMD act gradually; hence, both types of NMD could be used for homeostatic control of NMD intensity. Moreover, it might allow the partial separation of autoregulatory circuits of long 3′UTR-based and intron-based NMD systems.

## SUPPLEMENTARY DATA

Supplementary Data are available at NAR Online: Supplementary Table 7, Supplementary Figures 2–6 and Supplementary Data S1.

## FUNDING

Hungarian Scientific Research Fund [OTKA K60102, C77086]; International Centre for Genetic Engineering and Biotechnology [ICGEB CRP/HUN09-01]. T.N. and L.S. were graduate students of the ELTE ‘Classical and Molecular Genetics’ PhD program. EMBO short term fellowship program (to T.N.) and the Marie-Curie [PIEFGA-2009-253075] fellowship program (to Z.M.). Funding for open access charge: Grants from Hungarian Scientific Research Fund [OTKA K60102, C77086 and ICGEB CRP/HUN09-01] will be used to pay for the publication charge.

*Conflict of interest statement.* None declared.

## Supplementary Material

Supplementary Data
