# Supplementary material for: Plant nonsense-mediated mRNA decay is controlled by different autoregulatory circuits and can be induced by an EJC-like complex
Source: Nucleic Acids Res. 2013 May 10;41(13):6715–28. doi: 10.1093/nar/gkt366 (PMC3711448; doi:10.1093/nar/gkt366)
Supplement: Supplementary Data [file supp_41_13_6715__index.html]

Plant nonsense-mediated mRNA decay is controlled by different autoregulatory circuits and can be induced by an EJC-like complex — Plant nonsense-mediated mRNA decay is controlled by different autoregulatory circuits and can be induced by an EJC-like complex — Supplementary Data 

# Plant nonsense-mediated mRNA decay is controlled by different autoregulatory circuits and can be induced by an EJC-like complex

## Supplementary Data

files

**Files in this Data Supplement:**

- Supplementary Data - pdf file
