# Supplementary material for: Plant nonsense-mediated mRNA decay is controlled by different autoregulatory circuits and can be induced by an EJC-like complex
Source: Nucleic Acids Res. 2013 May 10;41(13):6715–28. doi: 10.1093/nar/gkt366 (PMC3711448; doi:10.1093/nar/gkt366)
Supplement: Supplementary Data [file supp_gkt366_nar-00302-a-2013-File008.pdf]

Supplementary data

S1. Details of clonings.

To clone G-30I, G-40I, G-50I, G-60I, G-70I and G-80I reporter constructs, pFF2R reverse and 30cF, 40cF, 50cF, 60cF 70cF and 80cF forward primers were used to PCR amplify the corresponding fragments from G-95I plasmid, and then these PCR products were cleaved and cloned into BamHI/SalI digested pBin-GFP vector. To generate G-95I, a PCR fragment was amplified from G-200I vector with 95cF and M13 F primers, cleaved and cloned into BamHI/EcoRI digested pBin-GFP vector. The G-95 control construct was created by RT-PCR with GFP start F and pFF2R primers from a mRNA sample that was isolated from G-95I infiltrated leaves. The RT-PCR fragment was cloned into BamHI/SalI digested pBin-mGFP vector.

To test whether 3'UTR introns can trigger intron-based NMD in heterologous 3'UTR context, different 3'UTR introns were PCR amplified from the *Arabidopsis* genome, and then these PCR products were cloned into XbaI digested G-95 vector. The orientation of the cloned fragments was determined by PCR. The selected clones were sequenced. SMG7 intron 6 and 7 were amplified with SMG7i6F/SMG7i6R and with SMG7i7F/SMG7i7R primer pairs, respectively. The $Barentsz_1$ intron 10 PCR fragment was generated with the B1i10F/B1i10R primers, the eRF1-1 intron 2 PCR product was amplified with the RF1-1iF/RF1-1iR primers and the eRF3 PCR fragment was produced with the RF3iF/RF3iR primers.

To clarify whether the putative EJC components are required for plant NMD, VIGS vectors containing a segment from one of the EJC component were generated. To clone TRV-P-Barentsz (TRV-P-Ba.) and TRV-P-4A3 clones, ~600 nt long exonic sequences were amplified from *N. benthamiana* cDNA with NbBar VIGSF/NbBar VIGSR and with Nb4A3 VIGS F/Nb4A3VIGS R primer pairs. The PCR fragments were cleaved and cloned into EcoRI digested TRV-PDS vector.

To analyse the role of PYM in plant NMD, agroinfiltration constructs expressing the full length PYM protein or its C-terminal or N-terminal deletion versions were created. To clone the PYM construct, an RT-PCR product generated from *N. tabaccum* cDNA with Nt PYM F and Nt PYM R primers was cleaved with BamHI/EcoRI, and then the digested PCR fragment was cloned into pBIN-Sanyi vector. PYM-N and PYM-C constructs were generated from PYM with Nt PYM F/Nt PYM N R and with Nt PYM C F/ Nt PYM R primer pairs. The PCR fragments were digested and cloned into BamHI/EcoRI cleaved pBIN-Sanyi vector.

To clarify whether the 3'UTRs of Barentsz$_1$ and SMG7 transcripts can induce NMD, Barentsz$_1$ and SMG7 terminator test constructs were generated. To create G-B$_1$T construct *Arabidopsis* Barentsz$_1$ (At1g80000) terminator region (the sequence from the stop to ~200 nt downstream from the polyadenylation site) was PCR amplified from genomic DNA using B1-T-F /B1-T-R primer pairs, and then the PCR fragment was cloned into a BamHI/EcoRI cleaved Bin-GFP vector. To generate G-B$_1$nospli.T, an unsplicable mutant version of the G-B1T construct in which the splicing acceptor site is impaired, PCR mutagenesis was performed using B1-T-F/ B1-T-3'ss mutR G-B1 and B1-T-3'ss mutF/B1-T-R primer pairs. The mutagenized PCR fragment was cleaved and cloned into BamHI/EcoRI digested Bin-GFP vector.

To study the role of the two 3'UTR introns in the SMG7 NMD regulation, mutants lacking intron 6 (G-S7Noi.6T) or intron 7 (G-S7Noi.7T) were generated by PCR mutagenesis from the G-S7T construct. To generate mutated PCR fragment lacking intron 6, G-F/SMG7-T-6idel-R and SMG7-T-6idel-F/SMG7-T-R primer pairs were used. To create mutant PCR product lacking intron 7, G-F/SMG7-T-7idel-R and SMG7-T-7idel-F/SMG7-T-R primer pairs were used. To generate a construct lacking both introns (G-S7Noi.T) PCR mutagenesis was carried out on G-S7Noi.6T construct using G-F/SMG7-T-7idel-R and SMG7-T-7idel-

F/SMG7-T-R primers. The three mutant PCR fragments were cleaved and cloned into BamHI/EcoRI digested Bin-GFP vector.

For Luc northern blot assays, PCR fragments were generated with JK.Luc F and JK.Luc R primers.

Primers used for cloning

**30cF**	CATGGATTCATCCTCTAGAAGATCTTATCC
**40cF**	CATGGATCCCTCAACCAAATCCTCTAGAAG
**50cF**	CATGGATCCGCCACCTTCGCCCTCAACCAA
**60cF**	CATGGATCCTTGAATCTCGCCAACTTCGCC
**70cF**	CATGGATCCTCTGAAGCTTTGAATCTCGCC
**80cF**	CATGGATCCTGGCACCACATCTGAAGCTTTGA
**95cF**	GAGCGTTCTTCCCGAGTG
**pFF 2 R**	AAT TCC CTT ATC TGG GAA CTA CTC AC
**GFP start F**	ATAACAATGAGTAAAGGAGAAGAACTT
**B1i10F**	ACAGTCTAGAATGAAGCCGTTTGGGC
**B1i10R**	TCTGTCTAGAACGGCTCAAGCTCAAG
**RF1-1iF**	ACA GTC TAG AAG CAT CTT TTG TGA AGG TA
**RF1-1iR**	ACA GTC TAG ACC ACA TGT TCC TTC ATT CATC
**RF3iF**	ACA GTC TAG AGG ATC CTG TGA GAG AAA AGG T
**RF3iR**	ACA GTC TAG AGT GCA ACC CGA AAG CCG TCT
**SMG7i6F**	CACTTCTAGAGATCAGAATGGAAGAGTA
**SMG7i6R**	CACTTCTAGAGTTACCACATATCTCTTCACCA
**SMG7i7F**	ACA GTC TAG AGC ACT TCC TAC CTG ACG GGT

**SMG7i7R**	ACA GTC TAG ACT CAC CTG CAT CAT TCC CAA
**Nb Bar VIGS F**	CAT GAA TTC CCT CTT GTG TGC CAC TGT A
**Nb Bar VIGS R**	TAG GTG AGG AGG ATG AAG TAG T
**Nb 4A3 VIGS F**	CAT GAA TTC GAG CCG TGG TTT TAA GGA TCA GA
**Nb 4A3VIGS R**	CAC GAA TTC CTG ACT CGG AAT CAA TCT TAG C
**Nt PYM F**	CAT GGA TCC ATG GCG ACC AGA GGA GGA GAA GA
**Nt PYM R**	CAT GAA TTC TTA AAC TAC GAG TTT CTT CAG CCT
**Nt PYM C F**	CATGGATCCGAGTCATTGCAGGATGTTCCGCCG
**Nt PYM N R**	CATGAATTCTCAAATGTCCTGCACAATATCTCCCAC
**B1--T-F**	ACTC GGA TCC GAC TAA GTG GAG TGA AAG GAG G
**B1-T-R**	GAA TTC AAC CAG AGC GGA GGC ATG TCT T
**B1-T-3’ss mut F**	GTTTTCCCGAGGTTGTTGTGCAATTG
**B1-T-3’ss mutR**	CAATTGCACAACAACCTCGGGAA
**SMG7-T-F**	CACT GGA TCC GAT CAG AAT GGA AGA GTA ACA CG
**SMG7-T-R**	CACT GAA TTC ATC GAA TCC TCT GCT GGG GTA TT
**SMG7-T-6idel-F**	GTA ATT GGG AAT GAT GCA GGT GAG TGT
**SMG7-T-6idel-R**	GCA TCA TTC CCA ATT ACC GTG TTA CTC TTC CAT TCT GA
**SMG7-T-7idel-F**	ATT CAG GAC ACA GCT CAT GTT G
**SMG7-T-7idel-R**	AGC TGT GTC CTG AAT CCG TCA GGT AGG AAG TGC CCC AC
**G-F**	CAT GGT CCT TCT TGA GTT TGT AAC AG
**S7 R2**	AGT AGC ACG AGA AAG GCA CT
**B_1R**	ACGCCCACGGCTCAAGCTCAA
**B_2R**	AGCCACTACGTCCCTTCTCC

**S7F**	CACT GGA TCC GAT CAG AAT GGA AGA GTA ACA CG
**B_1F**	ACTC GGA TCC GAC TAA GTG GAG TGA AAG GAG G
**B_2F**	CTCA GGA TCC TTT AGG GAC ACC TTG GCG CAG G
**JK.Luc F**	GGGTTACCTAAGGGTGTGGC
**JK.Luc R**	TCGCCTCTCTGATTAACGCC

Primers used for qRT-PCR assays

**At UBi F qRT-PCR**	TGC AAC CTC TC AAG TTC GAT TT TG
**At UBi R qRT-PCR**	CAA GCA GGA CTC CAA GCA TTC TTC A
**At Bar1 F qRT-PCR**	CCA ACC AGA ACA TGG TAC CGG AAA T
**At Bar1 R qRT-PCR**	CCC TGG TTT GTC TGC TTG GTA AGA A
**At Bar2 F qRT-PCR**	GGT CTG GCT CCA TTC GAT TTC ATT G
**At Bar2 R qRT-PCR**	GGG ATC ACT CTC ATA TTC CGC TTC A
**At SMG7 F qRT-PCR**	TGC CCG TGA CAA CTT GAT TGT TG
**At SMG7 R qRT-PCR**	GCT ACC AAG GTC GCA TCT TTC AAT G

Ubiquitin (At5g25760, Ubi) was used as a control for qRT-PCR studies,

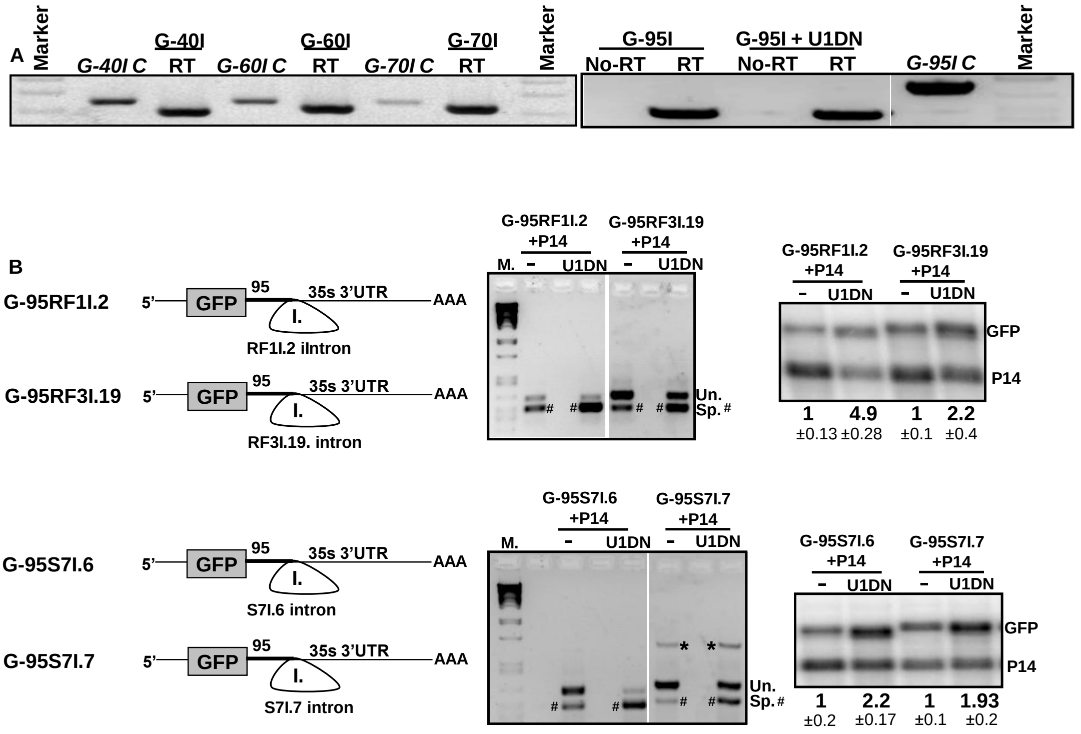

S2. Splicing of different introns located more than 50 nt downstream from the stop codon induces intron-based NMD. **(A)** The Ls coding region intron is spliced efficiently from the G-40I, G-60I, G-70I and the G-95I NMD reporter transcripts. Co-infiltration of the dominant-negative mutant of UPF1 (U1DN) does not modulate the efficacy of splicing of the Ls intron from G-95I mRNA. **(B-C)** Genuine 3'UTR introns can also trigger intron-based NMD. The 3'UTR introns of the putative *Arabidopsis* eRF1-1 intron 2 (RF1I.2), eRF3 intron 19 (RF3I.19) and SMG7 intron 6 and 7 (S7I.6 and S7I.7) were cloned 95nt downstream from the GFP (the constructs are referred as G-95RF1I.2, G-95RF3I.19, G-95S7I.6 and G-95S7I.7, respectively), and the efficiency of splicing and NMD sensitivity of the reporter transcript were studied in U1DN co-infiltration assays. Left panel: schematic, non-proportional representation of the reporter transcripts. Right panel: RT-PCR assays show that the RF1I.2, RF3I.19, S7I.6 and S7I.7 introns were only partially spliced in this heterologous context and that the spliced transcripts (marked #) are overexpressed in the U1DN co-infiltrated samples.

Thus the spliced mRNAs are targets of NMD. In line, G-95RF1I.2, G-95RF3I.19, G-95S7I.6 and G-95S7I.7 reporter transcripts accumulate to enhanced levels in U1DN co-infiltrated samples.

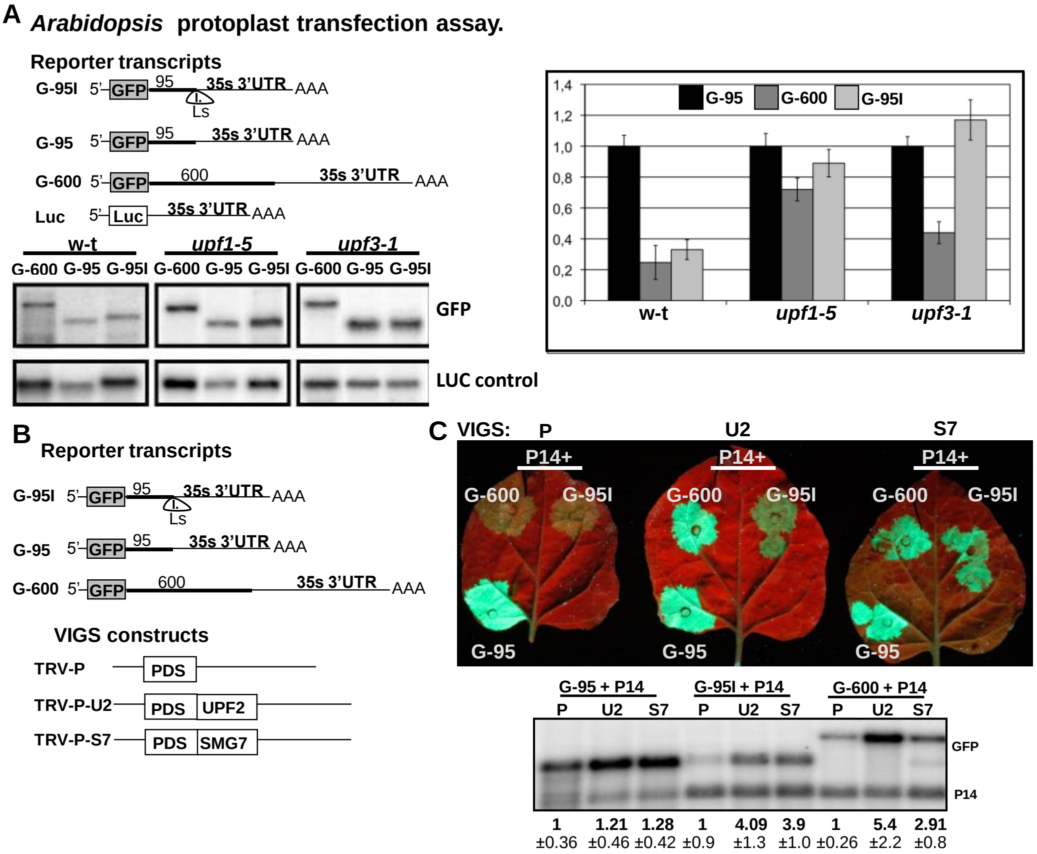

S3. UPF3, UPF2 and SMG7 are involved in both types of NMD. **(A)** Both types of NMD are impaired in the protoplasts of *upf3-1* NMD mutant *Arabidopsis* plants. Left, upper panel: schematic, non-proportional representation of the reporter constructs that were used for protoplast transfection assays. Protoplast isolated from the leaves of wild-type (w-t) and NMD mutant (*upf1-5* and *upf3-1*) *Arabidopsis* plants were co-transfected with two plasmids, a normalization control plasmid expressing luciferase (luc) transcript and a plasmid that expressed the G-95 control mRNA or the G-600 long NMD reporter or the G-95I intron-based NMD reporter transcripts. RNA gel blot assays were carried out from total RNAs isolated at 1 d.p.i. After normalization the G95 value was taken as 1. *upf1-5* is a hypomorphic mutant, while *upf3-1* might be a null mutant. In w-t control protoplasts, both NMD reporter transcript accumulate to low levels relative to the G-95 control, indicating that both types of NMD function in *Arabidopsis* leaf protoplast. Note that in *upf1-5* mutant the control G-95 and the G-600 and G-95I NMD reporter transcripts express to comparable levels. By

contrast, in *upf3-1* mutant protoplasts the G-600 mRNA accumulates to relatively low levels, while the G-95I reporter and the G-95 control transcripts accumulate to comparable levels. These data indicate that UPF1 is essential for both types of NMD and that UPF3 is required for intron-based NMD. Apparently, UPF3 enhances the efficacy of long 3'UTR-based NMD but it is not essential for long 3'UTR-based NMD. **(B)** Schematic, non-proportional representation of the constructs that were used for VIGS-agroinfiltration assay. TRV-P, TRV-P-U2 and TRV-P-S7 VIGS construct were used to silence the control (PDS) and the test genes (UPF2 and SMG7, respectively). G-600 is the long 3'UTR-based NMD reporter, G-95I is the intron-based NMD reporter and G-95 is a non-target negative control construct. The NMD reporters and the control are shown as transcripts. **(C)** UPF2 and SMG7 are required for both long 3'UTR- and intron-based NMD. Both NMD reporters are overexpressed in UPF2-, and SMG7-silenced plants. Leaves of PDS-silenced (P) negative control and the leaves of the UPF2-silenced (U2) and the SMG7-silenced (S7) test plants were co-infiltrated with P14 and the G-600 or the G-95I NMD reporters or with the G-95 control constructs. RNA gel blots were hybridized with GFP and P14 probes, and then samples were quantified as described at Figure 1.

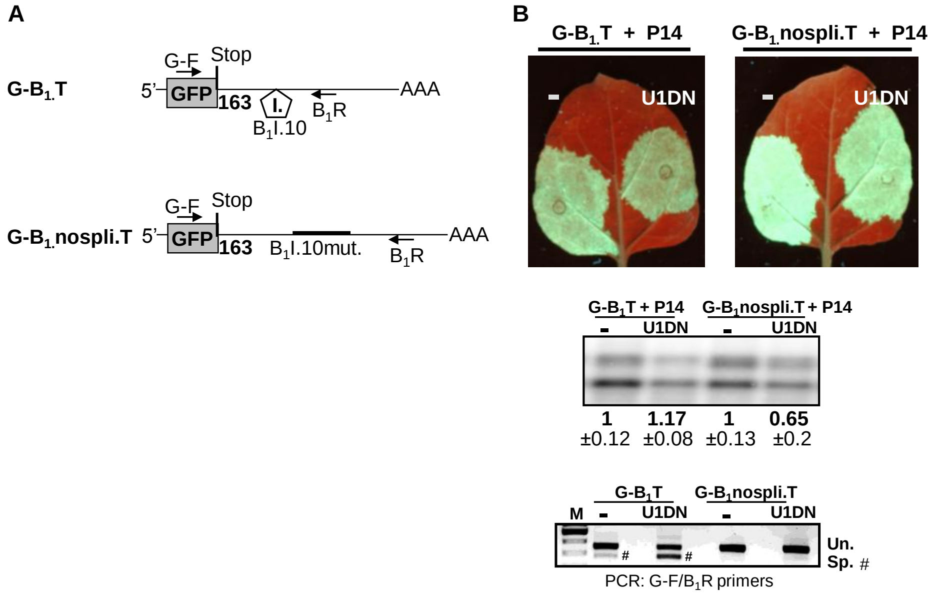

S4. Splicing of the 3'UTR located Barentsz1 intron 10 (B_1I.10) induces intron-based NMD in heterologous context. **(A)** Schematic, non-proportional representation of the reporter transcripts. The terminator region of Barentsz1 (from stop to 200 nt downstream of the polyadenylation site) was cloned downstream of the GFP reporter gene (G-B_1T). As a control, a non-spliceable control was created (G-B_1nospli.T). **(B)** G-B_1T and G-B_1nospli.T reporter constructs were infiltrated with P14 (-) or were co-infiltrated with P14 and a dominant-negative mutant of UPF1 (U1DN), and then RNA gel blot and RT-PCR assays were conducted. Note that the same RT-PCR assay is also shown at Figure 5C.

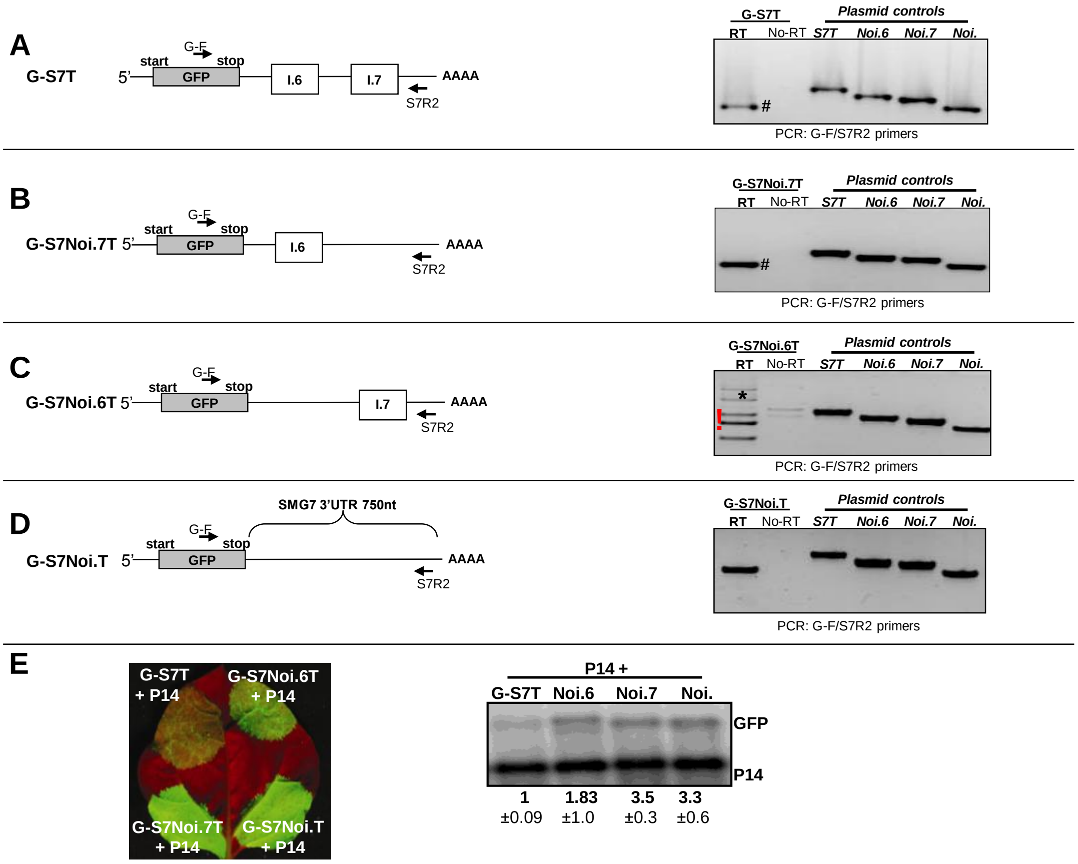

S5. Splicing of the 3'UTR located introns of SMG7. **(A-D)** The *Arabidopsis* SMG7 terminator was cloned downstream of GFP (G-S7T), and then three different deletion constructs were generated to study the splicing of the two 3'UTR introns. In the three deletion mutant constructs the distal intron (G-S7Noi.7T), the proximal intron (G-S7Noi.6T) or both introns were deleted (G-S7Noi.T). Schematic, non-proportional representation of the reporter transcripts (**A-D** left panels). RT-PCR assays were conducted to study the splicing of the introns. Control PCRs generated from the plasmids with the same primers are shown at the right side of each gel (Shortened plasmid names are given in italics. G-S7T is shown as S7T, G-S7Noi.7T as Noi.7, G-S7Noi.6T as Noi.6 and G-S7Noi.T as Noi, respectively).

Note that both introns are efficiently spliced from G-S7T transcript (**A**, right panel, # marks the spliced transcript.) and that the stop proximal S7I.6 intron is efficiently spliced from G-S7Noi.7 transcripts lacking the distal intron (**B**, right panel #). Thus the distal intron is not required for the splicing of the stop proximal intron. By contrast, the distal intron (S7I.7) was

inefficiently and aberrantly spliced (marked by a red exclamation mark) from the reporter transcript (G-S7Noi.6T) lacking the stop proximal I.6 (**C**, right panel). Sequencing revealed that S7I.7 intron was sometimes correctly spliced from G-S7Noi.6T transcripts but alternative splicing products were also detected. Unexpectedly, transcripts that were longer (*) than the unspliced mRNAs, were also observed. These transcripts might be alternative cleavage/polyadenylation products. **(E)** The expression of the different GFP-SMG terminator mutants. G-S7T, G-S7Noi.6T, G-S7Noi.7T and G-S7Noi. reporter constructs were agroinfiltrated and their expression was analyzed at 3 d.p.i.

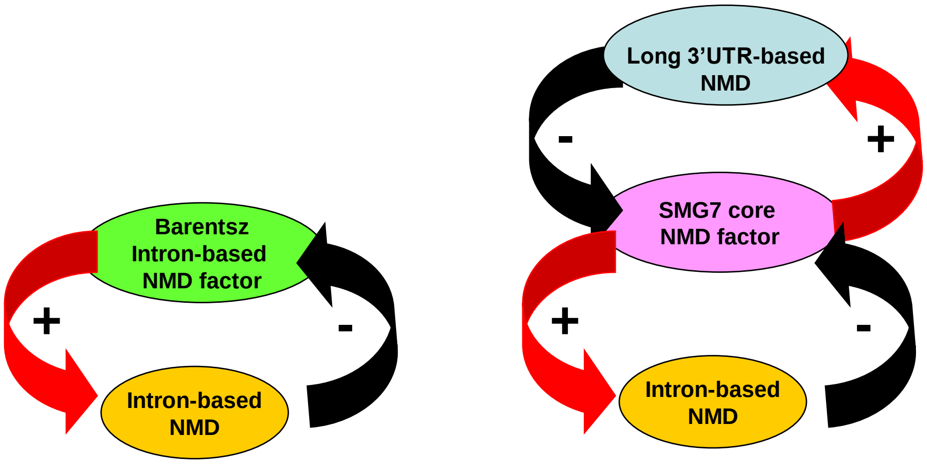

S6. Plant NMD is controlled by two autoregulatory circuits. The expression of the mRNA of the SMG7 NMD factor that is required for both types of NMD is negatively regulated by the long 3'UTR-based as well as the intron-based NMD systems. The expression of the mRNA of Barentsz intron-based NMD factor is downregulated by intron-based NMD. The model proposes that the intensity of intron-based NMD can be modulated by altering the expression of SMG7 and/or Barentsz, while the intensity of long 3'UTR-based NMD only depends on the expression of SMG7.

S7. The 3'UTR located NMD features of SMG7 and Barentsz mRNAs are conserved within angiosperms.

(A) Summarizing tables: the NMD features of the 3'UTR of annotated angiosperm SMG7, SMG7L and Barentsz transcripts (upper, middle and bottom tables, respectively).

Intron-based NMD sensitive 3'UTRs contain at least one NMD relevant intron. Long 3'UTR-based NMD sensitive 3'UTRs are longer than 500 nt. Long 3'UTR- and intron-based NMD sensitive 3'UTRs are longer than 500 nt and contain at least one NMD relevant intron.

3'UTR introns located more than 50 nt downstream from the stop codon are referred to as NMD relevant, whereas introns located less than 50 nt downstream from the stop are referred to as NMD irrelevant introns. 3'UTR data were collected from Phytozome database.

(B) The NMD features of the 3'UTRs of the annotated angiosperm SMG7 transcripts. Relevantly, almost all SMG7 transcripts are sensitive to both types of NMD. If alternative splicing is documented in the 3'UTR, only one transcript (main transcript) is coloured according the colour code, while the alternative splicing isoforms are shown in yellow. In the summarizing table only the main transcript was calculated. Red letters show the position of NMD relevant and black letters show the position of NMD irrelevant introns. The intron positions of the main transcripts are given in bold. **(C)** The 3'UTRs of the annotated SMG7L transcripts do not contain NMD features. SMG7L is a dicot specific paralog of SMG7. Indirect evidence suggests that SMG7L is not required for NMD. In line, NMD *cis* elements could not be identified in the 3'UTR of any annotated SMG7L transcripts. **(D)** The NMD features of the 3'UTRs of the annotated angiosperm Barentsz transcripts. Note that Barentsz is present in multiple copies in most completely sequenced angiosperms. Dicot Barentsz mRNAs are potentially regulated by intron-based NMD or by both types of NMD, while monocot Barentsz transcripts might be sensitive to both types of NMD. Relevantly, in average the 3'UTRs of SMG7 transcripts are longer than the 3'UTRs of Barentsz mRNAs (799 and 547 nt, respectively). Thus the transcripts of the EJC component Barentsz are less sensitive to long 3'UTR-based NMD than the mRNAs of the core NMD factor SMG7.

S7A: The 3’UTR located NMD features of SMG7 and Barentsz mRNAs are conserved within Angiosperms

The NMD features of the 3’UTRs of the annotated Angiosperm SMG7 transcripts

			**Long 3’UTR- and intron-based NMD sensitive 3’UTR**		**Long 3’UTR-based NMD sensitive 3’UTR**	**NMD insensitive 3’UTR**
	Number of different SMG7 transcripts (Number of species with annotated SMG7 3’UTR)	Average length of SMG7 3’UTR	Long 3’UTR with 2 introns (1 NMD irrelevant +1 NMD relevant)	Long 3’UTR with only NMD relevant intron(s)	Long 3’UTR without NMD relevant intron	Short 3’UTR without NMD relevant intron
**Monocots**	6 (6)	836	6	0	0	0
**Dicots**	29 (20)	791	24	2	3	0
**Angiosperm**	35 (26)	799	30	2	3	0

All SMG7 transcripts have NMD sensitive 3’UTR: 30/35 transcripts have 1 NMD irrelevant and 1 NMD relevant intron, 2/35 contain 1 or 2 NMD relevant introns but do not have NMD irrelevant intron and 3/35 have long, intronless 3’UTR. No annotated SMG7 transcript has only intron-based NMD sensitive 3’UTR.

The 3’UTRs of the annotated SMG7L transcripts do not contain NMD features

			**Intron-based NMD sensitive 3’UTR**	**Long 3’UTR-based NMD sensitive 3’UTR**	**NMD insensitive 3’UTR**
	Number of different SMG7L transcripts (Number of species with annotated SMG7L 3’UTR)	Average length of SMG7L 3’UTRs			
**Dicots**	11 (11)	219	0	0	11

The NMD features of the 3’UTRs of the annotated Angiosperm Barentsz transcripts

			**Long 3’UTR- and intron-based NMD sensitive 3’UTR**<br>Long 3’UTR with at least one NMD relevant intron	**Intron-based NMD sensitive 3’UTR**<br>Short 3’UTR with NMD relevant intron	**Long 3’UTR-based NMD sensitive 3’UTR**<br>Long 3’UTR without NMD relevant intron	**NMD insensitive 3’UTR**<br>Short 3’UTR without NMD relevant intron
	Number of different Barentsz transcripts (Number of species with annotated Barentsz 3’UTR)	Average length of Barentsz 3’UTRs				
**Monocots**	14 (6)	529	11	0	0	3
**Dicots**	28 (17)	549	17	10	0	1
**Angiosperm**	42 (23)	547	28	10	0	4

38/42 Barentsz transcripts contain one or more NMD relevant intron(s), all monocot (11) and 17 dicot Barentsz mRNA have long 3’UTR with NMD relevant introns,while 10 dicot Barentsz transcripts have exclusively intron-based NMD sensitive 3’UTR. 4/42 have NMD insensitive 3’UTR and none of them is exclusively sensitive to the long 3’UTR-based NMD.

S7B:The NMD features of the 3’UTRs of the annotated Angiosperm SMG7 transcripts

Long 3’UTR- and intron-based NMD sensitive 3’UTR (light orange)
Long 3’UTR-based NMD sensitive 3’UTR (yellow)
Intron-based NMD sensitive 3’UTR (cyan)
NMD insensitive 3’UTR (magenta)
Alternative splicing in the 3’UTR (AS) (purple)

			**Long 3’UTR- and intron-based NMD sensitive 3’UTR**			**Long 3’UTR-based NMD sensitive 3’UTR**	**Intron-based NMD sensitive 3’UTR**	**NMD insensitive 3’UTR**
			Long 3’UTR with 2 introns (1 NMD irrelevant and 1 NMD relevant)		Long 3’UTR with only NMD relevant intron(s)	Long 3’UTR without intron	Short 3’UTR with NMD relevant intron	Short 3’UTR without NMD relevant intron
	Species with annotated SMG7 3’UTR	Length of SMG7 homolog 3’UTRs	Distance of NMD irrelevant intron from the stop	Distance of NMD relevant intron from the stop	Distance of NMD relevant intron from the stop			
**Monocots**	Sorghum bicolor	1176	29	167				
	Zea mays	720	29	167				
	Setaria italica	793	29	170				
	Panicum virgatum	793	29	170				
	Oryza sativa	701	18	157				
	Brachypodium distachyon	836	27	180				
**Dicots**	*Manihot esculenta*	701						
	Ricinus communis	823	19	166				
	Gossypium raimondii	671	10	64				
		668	10	150				
		654	21	160				
		795						
	Populus trichocarpa	**1113**	**21**	**158**				
		1213			256			
		AS 1330						
	Medicago truncatula	504	21	124				
	Phaseolus vulgaris	650	28	162				
		806	21	145				
	Glycine max	716	22	146				
		763	22	146				
		801	40	161				
	Theobroma cacao	**595**	**21**	**160**				
		AS 258	22	160				
	Cucumis sativus	**743**	**21**	**151**				
		AS 843			251			
	Prunus piersica	641	18	157				
	A.thaliana	579	23	158				
	A.lyrata	544	26	156				
	Capsella rubella	794			227			
	Thellungiella halophila	476	23	139				
	Citrus siensis	2244						
	Citrus celementina	853	20	167				
	Eucalyptus grandis	1443			560 and 694			
	Mimulus guttatus	795	26	147				
		605	23	158				

	Aquilegia coerula	795	30	209				
		838	30	209				
	Vitis vinifera	785	24	194				
		560	21	153				

S7C:The 3'UTR of the annotated SMG7L transcripts do not contain NMD features

	Species with annotated SMG7L 3'UTR	Length of SMG7L homolog 3'UTRs	Long 3'UTR-based NMD sensitive 3'UTR	Intron-based NMD sensitive 3'UTR	NMD insensitive 3'UTR
**Dicots (11)**	*Populus trichocarpa*	361			
	Medicago truncatula	329			
	Phaseolus vulgaris	151			
	Theobroma cacao	250			
	Glycine max	131			
	Cucumis sativus	167			
	A.thaliana	176			
	Thellungiella halophila	205			
	Citrus siensis	262			
	Citrus clementina	262			
	Aquilegia coerula	119			

S7D:The NMD features of the 3'UTRs of the annotated Angiosperm Barentsz transcripts

Long 3'UTR- and intron-based NMD sensitive 3'UTR
Long 3'UTR-based NMD sensitive 3'UTR
Intron-based NMD sensitive 3'UTR
NMD insensitive 3'UTR
Alternative splicing in the 3'UTR (AS)

				Long 3'UTR- and intron-based NMD sensitive 3'UTR			Long 3'UTR-based NMD sensitive 3'UTR	Intron-based NMD sensitive 3'UTR	NMD insensitive 3'UTR
	Species with annotated Barentsz 3'UTR	Length of Barentsz 3'UTR		Distance of 1. intron from the stop	Distance of 2. intron from the stop	Distance of 3. intron from the stop	Long 3'UTR without (NMD relevant) intron	Short 3'UTR with NMD relevant intron(s)	Short 3'UTR without (NMD relevant) intron
**Monocots**	*Sorghum bicolor*	559		151	234				
		722		42	120	202			
	Zea mays	662		42	120	201			
		706		140	121				
		263							
	Setaria italica	716		142	232				
		516		42	120				
	Panicum virgatum	121		42					
		501		44	120	202			
		550		144	227				
	Oryza sativa	479		13					
		723		144	227				
	Brachypodium distachyon	563		45	124	211			
		AS	**626**	**158**	**279**				
			240						
**Dicots**	*Manihot esculenta*	496		32	110				
		595		34	113	202			
	Ricinus communis	586		35	114	203			
	Gossypium raimondii	493		37	112	205			
		880		38	119	211			
		787		42	118	207			
	Populus trichocarpa	562		34	113				
		741		18	245				
	Phaseolus vulgaris	217		41	131				
		455		35	89	203			
		520		35	109	165			
	Glycine max	AS	**606**	**41**	**117**	**187**			
			530	41	111				
		AS	**775**	**38**	**118**				
			694	38	118	202			
	Theobroma cacao	610		43	124	213			
	Cucumis sativus	641		43	121	203			
	A. thaliana	338		163					
		451		118					
	A. lyrata	218		154	176				
		424		120	213				
	Capsella rubella	363		110					
	Thellungiella halophila	186							
		487		116					
	Citrus siensis	254		5	211				
		692		185	274				
	Citrus clementina	AS	**750**	**185**	**274**				
			674	30	109	198			
	Eucalyptus grandis	706		192	279				
	Mimulus guttatus	635		206	294				
	Aquilegia coerulea	AS	**813**	**87**					
			403						
